# Ultrasonic emulsification of *Cananga odorata* nanoemulsion formulation for enhancement of herbicidal potential

**DOI:** 10.1038/s41598-025-87810-1

**Published:** 2025-01-25

**Authors:** Chamroon Laosinwattana, Naphat Somala, Jantra Dimak, Montinee Teerarak, Nawasit Chotsaeng

**Affiliations:** 1 School of Agricultural Technology, King Mongkut’s Institute of Technology Ladkrabang, Bangkok, 10520 Thailand; 2 School of Science, King Mongkut’s Institute of Technology Ladkrabang, Bangkok, 10520 Thailand

**Keywords:** Natural herbicide, Herbicidal activity, Ultrasonication, Emulsification method, Plant sciences, Nanoscience and technology

## Abstract

A nanoemulsion was fabricated from *Cananga odorata* essential oil (EO) and stabilized by incorporation of Tween 80 using ultrasonication. The major constituents of the EO were benzyl benzoate, linalool, and phenylmethyl ester. Differing sonication amplitude (20–60%) and time (2–10 min) were assessed for effects on nanoemulsion droplet size and polydispersity index (PI). The smallest droplet size of 43.98 nm (PI 0.222) was obtained using 40% amplitude for 8 min; this nanoemulsion was evaluated for its droplet characteristics and pre-emergence herbicidal activities on *Amaranthus tricolor*. FT-IR confirmed ultrasonic emulsification to not affect the EO components. Regarding stability, storage at 4 °C was determined appropriate, with droplet size changing slightly after five weeks. Assays of herbicidal potential showed the coarse emulsion and nanoemulsion to both reduce *A. tricolor* germination and growth, with the nanoemulsion being more effective at a given concentration and the difference in effectivity correlating to droplet size. Remarkably, treatment with 250 ppm nanoemulsion and coarse emulsion respectively resulted in 100% and 63.75% germination inhibition. Both emulsions decreased seed imbibition and alpha-amylase activity. The highest relative electrolyte leakage was achieved in seed treated with the nanoemulsion. Therefore, this ultrasonic-based nanoemulsion may have utility as bioherbicide alternative.

## Introduction

Nanotechnology, in which controllable particle sizes range 10–200 nm, is the most advanced technology of the 21st century so far^1,2^. Agriculture urgently needs innovation to meet the rising demand for food while simultaneously minimizing impacts on the environment. Nano-herbicides have great potential to lower the amount of pesticides used and boost their efficacy^3^. Utilizing nano-formulations makes it possible to apply the minimal effective concentration of the active ingredients at target areas and to deliver the active ingredients gradually into a plant’s root zone or its internals without sacrificing efficacy^4^.

Essential oils (EOs) are concentrated, volatile substances extracted from plants, characterized by their natural aroma and composed of a mixture of various chemical compounds that are known to have natural herbicidal properties due to their allelochemical composition^5,6^. When EOs are used as bioherbicides, their mode of action may include disruption of cell membranes, increased oxidative stress, suppression of seed germination, and certain metabolic inhibitory mechanisms. EOs represent less of a concern to human health when disposed of in environmental matrices since they biodegrade more quickly than traditional agrochemicals^7^. *Cananga odorata* Hook. F. & Thomson (Family: Annonaceae) (ylang-ylang) is a native Indian tree with rapid growth^8^ and reported antibacterial, antifungal, and cytotoxic activities^9^. *C. odorata* EO has been observed to have pre-emergence herbicidal action^10^. However, the biological activity and applicability of EOs are limited by their high volatility, fast breakdown, and weak water solubility^11^. For use as herbicides, EO-based products have been formulated to produce coarse emulsions. According to previous reports, EO-based nanoemulsions have improved their biological activity, solubility, and storage duration^12^.

Nanoemulsion production techniques may be classified broadly based on the underlying physicochemical process of droplet disruption: either high-energy or low-energy approaches^13^. Ultrasonic emulsification is an efficient technique for the preparation of nanoemulsions with long-term stability^14–16^. Nanoemulsion encapsulation and ultrasonication also effectively improve the bioaccessibility, solubility, surface hydrophobicity, encapsulation rate, storage stability, and release control of bioactive components^2^. In the ultrasonic emulsification method, a two-step procedure, a macroemulsion is broken up by cavitation due to ultrasonic waves produced by a probe^16,17^. In the first step, an acoustic field is used to generate a mixture of interfacial waves. The instability of these waves causes the oil phase to explode into the aqueous medium as droplets. In the second step, cavitation caused by low-frequency ultrasonic waves breaks up the droplets^16,18,19^. Notably, ultrasonication is a nonthermal technology that is safe, noninvasive, nonionizing, effective, eco-friendly, and widely used in food engineering to assist in extraction with clean label foods^2^.

The non-ionic surfactant Tween 80 is able to adsorb on the surface of oil droplets with fast mobility and dense attachment. By reducing the surface free energy, the nonionic small molecule surfactants can efficiently pack at the interface^20^. Tween 80 can reduce interfacial tension between water and oil phases. The low dynamic interfacial tensions in Tween 80 formulations allow for the easy formation of small droplets^21^. Tween 80 possesses good stabilizing properties^22^, serving to stabilize emulsion droplets by stearic stabilization. Due to steric repulsion forces, nonionic surfactants adsorb at the particle surface, preventing other particles from approaching^23^. Moreover, because of its low molecular weight, it reduces droplet size to a greater degree than polymeric surfactants^15^. In addition, the large hydrophilic polyoxyethylene groups it contains may cause relatively significant spatial repulsion^24^. Stabilised nanoemulsions are promising carriers of EOs, as such solutions can increase the water solubility and bioavailability of the oils, prevent degradation of the encapsulated components, guarantee sustained release of the oils, and facilitate using less active ingredient to get the a desired result. Moreover, the biological activity of essential oils can be enhanced and their negative effects decreased through the utilization of nanoemulsions. A nanoemulsion formulation promotes the interaction of EO compounds with the various molecular sites at the cell membrane because of its small droplet size and increased surface area. Importantly, breakdown and increased permeability of cell membranes may underlie the herbicidal action of EO compounds^25^. In formulation of bioherbicides, low-viscosity nanoemulsions are essential because they enable aerosol application, with which pesticide-containing nanoemulsions settle uniformly on plant leaves^25^. In recent decades, there has been notable advancements in the production of nanoemulsions containing EOs^7^. Natural products intended for use as natural herbicides are developed into nanoemulsion forms that can improve the effectiveness of active ingredients and increase dispersion wettability^14,26^. Recently, the potential of nanotechnology was used for developing nanoherbicides as an effective alternative to traditional weed control methods. Nanotechnology can increase herbicide effectiveness by reducing dosage requirements and decreasing off-target effects, improving stability and bioavailability^27,28^. Chen et al.^29^ reported that Their EO-based nanoemulsion enabled it possible to reduce the EO dosage by 2–4 times while affecting the same level of weed-killing effectiveness. The outstanding enhancement attributed to the nanoemulsions improved resistance to wash-off, increased leaf retention, and improved leaf-wetting capabilities.

This study is the first to develop the biodegradable *C. odorata* EO-based nanoherbicide using the ultrasonication emulsification method for controlling the targeted *A. tricolor* weed plant. The main objective of this research was to prepare nanoemulsion from EO as a pre-emergence herbicide using ultrasonication technique for enhancing its effectiveness. *C. odorata* EO and Tween 80-based nanoemulsion was formulated by ultrasonic emulsification at a sonication amplitude of 20–60% for 2–10 min, optimizing sonication amplitude and time to generate the nanoemulsion with smallest droplet size. In addition, the optimized nanoemulsion was investigated for its physical characteristics and pre-emergence herbicidal activities.

## Materials and methods

### Plant and chemical materials

*Amaranthus tricolor* seeds were purchased from Chia Tai Co., Ltd. (Bangkok, Thailand). *C. Odorata* EO was obtained from Chemipan Corporation Co., Ltd. (Bangkok, Thailand). Tween 80 (hydrophile-lipophile balance 15) was purchased from Sigma-Aldrich. Deionized water was purchased from RCI Labscan (Ireland) and used in all experiments.

### Identification of *C. Odorata* EO constituents by gas chromatography/mass spectrometry (GC/MS)

Gas chromatography/Mass spectrometry (GC/MS) was used to identify the constituents of *C. odorata* EO. An Agilent 5973 mass detector was connected to an Agilent series 6890 N gas chromatograph equipped with a HP-5 silica capillary column (30 m; 0.25 mm ID; film thickness 0.25 μm). Operating parameters included an oven temperature of 40 °C (3 min); a temperature range of 10 to 100 °C (5 min); and a flow rate of 1 ml/min of helium gas. The sample (0.2 ml) was injected in split mode (1:50) into the capillary column. The temperature of the ion source and interface was 230 °C and 280 °C, respectively. Individual constituents were distinguished via comparison of their mass spectra (molecular mass and fragmentation pattern) with the internal reference mass spectra library (National Institute of Standards and Technology, NIST, 2014). The relative amount of each component was expressed as the percentage of overall peak area.

### Preparation of *C. Odorata* nanoemulsion

**Fig. 1:**
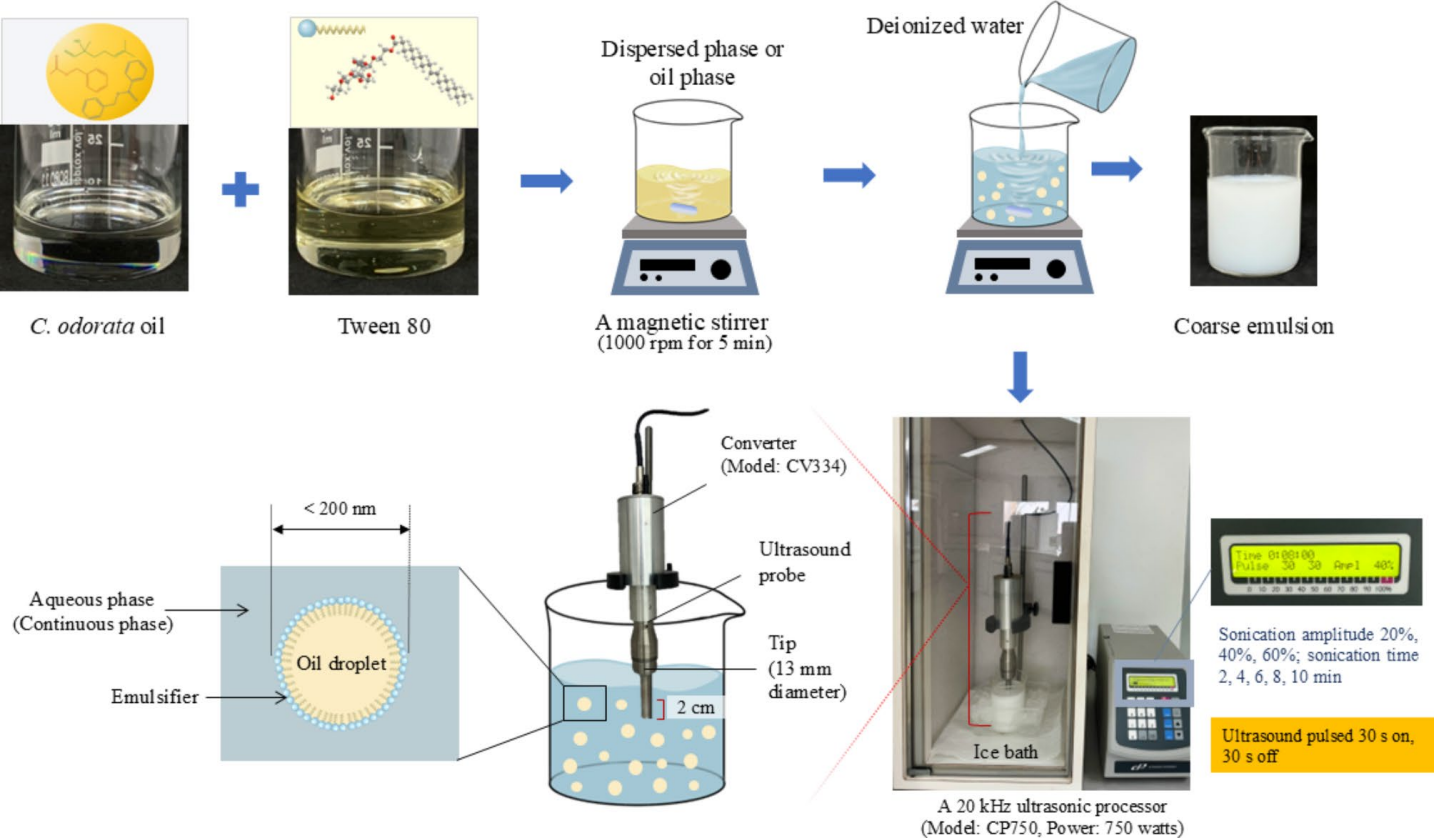
Schematic illustration of *C. odorata* nanoemulsion preparation using ultrasonication emulsification.

*C. odorata* nanoemulsions were prepared by a two-step process, wherein the initial step was the formation of a coarse oil-in-water (O/W) emulsion by mixing the three components (*C. odorata* EO, deionized water, and Tween 80 as emulsifier). Firstly, *C. odorata* EO (1% w/v) and Tween 80 (1% w/v) were mixed using a magnetic stirrer (AREX 5 Digital, VELP^®^ SCIENTIFICA, Italy) at 1000 rpm for 5 min to obtain the dispersed phase or oil phase. Deionized water was added to the obtained mixture and continually mixed for 5 min to yield the coarse emulsion (Fig. 1). The obtained *C. odorata* EO coarse emulsion, with a large droplet size, was used for nanoemulsion preparation.

Nanoemulsion formulations were then prepared from the coarse emulsion using a 20 kHz ultrasonic processor (Model: CP750, Power: 750 watts) from Cole-Parmer Instruments, Illinois, USA. An ultrasound probe with a tip of 13 mm diameter was used to generate disruptive forces. When the probe is dipped in a sample and ultrasonic waves emitted, the waves cause cavitation bubbles to form, which develop until they collapse^30^. To prevent overheating, the ultrasonic pulser was operated in intervals of 30 s on and 30 s off. The sample was kept in an iced water bath during processing to reduce the heat generated during sonication. Sonication parameters were varied, with amplitudes of 20%, 40%, and 60% and times of 2, 4, 6, 8, and 10 min. All experiments were performed in a 200 mL glass beaker. The obtained nanoemulsion formulations were stored at a temperature of 4 °C for further experiments. The selection of sonication amplitudes with time is an important parameter for the nanoemulsion preparation and optimization. The smallest droplet size was considered as a criterion for selecting the optimal nanoemulsion. Sonication amplitude with time influenced the energy input and the resulting breakdown of droplets and droplet size distribution.

### Characterization of nanoemulsions

#### Nanoemulsion droplet analysis

 Dynamic light scattering (DLS) with the Nanoplus 3, MICROMERITICS, Japan was employed to determine the average diameter droplet size and polydispersity index (PI) of the O/W nanoemulsion. Two mL of each treatment solutions were added into a cuvette. Measurement conditions consisted of a fixed angle of 165° and a temperature of 25 °C. The nanoemulsion samples were diluted with deionized water at a ratio of 1:9 to avoid multiple-scattering effects during droplet analysis. Five replications were performed for each measurement, and the program nanoPlus version 5.10/3.00 was used for computations. The nanoemulsion with the smallest droplet size was selected as the optimal formulation for further experiments.

#### Fourier-transform infrared (FT-IR) spectroscopic analysis

 To identify the functional groups and covalent contacts, if any, present in the pure EO, coarse emulsion, and optimal nanoemulsion, FT-IR spectra were collected over the wavenumber range of 4000–400 cm^− 1^ using an FT-IR spectrometer (Invenio-s, Bruker). Thirty-two scans at a resolution of 2 cm^− 1^ were recorded for each spectrum. A completely randomized design was used with four replications.

#### Morphology of the optimal nanoemulsion

The droplet shapes of the optimal nanoemulsion were investigated to confirm the calculated droplet size. A transmission electron microscope (HITACHI HT7700, Japan) operating at 80 kV was used to observe the morphology of the nanoemulsion. After allowing a drop of nanoemulsion to settle on a grid covered with carbon for ten minutes, a drop of 2% uranyl acetate was added, and the mixture was let stand for thirty seconds. A completely randomized design was used with four replications.

#### Rheological property and surface tension of the optimal nanoemulsion

Rheological properties of the optimal *C. odorata* nanoemulsion were determined by a MCR 302 Modular Compact Rheometer (Anton Paar) for the shear rate range of 0–250 s^− 1^ in 600 s. The rheometer was filled with 7 mL of the nanoemulsion. Rheological properties were performed using Rheostar program. A DY-300 surface tension meter (Kyowa Interface Science, Japan) was used to measure the surface tension of the nanoemulsion using the Wilhelmy plate method at a monitored temperature of 25 ± 1 °C.

#### Storage stability of the optimal nanoemulsion

A centrifugation test was performed to investigate the impact of gravity on the nanoemulsion using an MPW-260R (MPW, Poland) for 20 min at 10,000 rpm and at temperature of 25 °C. Ten mL of the optimal nanoemulsion was used for centrifugation test. Then, the nanoemulsion was kept for five weeks at 4 and 25 °C in a glass bottle without light. After preparation and centrifugation, the droplet size and PI value was measured at one-week intervals. A completely randomized design was used with five replications.

### Herbicidal activity

### Germination bioassay

Healthy, equal-sized seeds of *A. tricolor* were selected and used in Petri dish assays to evaluate seed germination and seedling growth inhibition by the *C. odorata* EO-based optimal nanoemulsion and coarse emulsion. Both treatments were prepared at concentrations of 62.5, 125, 250, and 500 ppm. Five mL of each treatment solution was added to Petri dishes with germination papers. Twenty seeds were placed onto each germination paper and the dish cover sealed. The dishes were kept in a growth chamber (LAC-1075-N, Longyue, Shanghai) (12/12 light/dark, at 25 ± 2 °C, humidity of 80%) for seven days. Distilled water and Tween 80 solution (500 ppm) served as controls. Germination count and root and shoot lengths (cm) were recorded after seven days of incubation. A completely randomized design was used, with four Petri dishes set up as replication for each treatment. Inhibition of germination and seedling length were both determined as percentages calculated with the following equation:

$${\text{Inhibition }}\left( {\% \;{\text{over }}\;{\text{control}}} \right)=\left[ 1 - \left( {{\text{sample}}\;{\text{ formulation}}/{\text{control}}} \right) \right] \times 100$$

#### Morphology

Micro-morphological seed traits were examined using a scanning electron microscope and energy dispersive x-ray spectrometer (model: JEOL JSM-IT500HR). *A. tricolor* seeds were treated with the optimal nanoemulsion and coarse emulsion at concentration of 250 ppm for 18 h. After incubation, the treated seeds were first fixed in 70% ethanol, then dehydrated using a series of increasing ethanol concentrations. Afterwards, the seeds were placed on metallic stubs using double adhesive tape, coated with gold for six minutes in a sputtering chamber, and finally examined. Various image magnifications were employed (ranging from 75× to 500×) depending upon the size of the seeds.

#### Seed imbibition and α-amylase activity

The optimal nanoemulsion and coarse emulsion were evaluated for seed imbibition and α-amylase activity. Treatment formulations consisted of each nanoemulsion at concentrations 250 and 500 ppm, and Tween solution at a concentration of 500 ppm. Water was used as a control. A completely randomized design was used with four replication.

The process of seed imbibition was carried out according to Turk and Tawaha^31^, with modification. Briefly, 100 *A. tricolor* seeds were weighed (W_1_) and soaked in the treatment solution for 6, 12, and 18 h. After incubation, the seeds were washed and re-weighed (W_2_). Seed imbibition percentage was then determined using the following equation:

$${\text{Seed }}\;{\text{imbibition }}\left( \% \right)=\left[ {\left( {{{\text{W}}_{{2}}}--{{\text{W}}_{{1}}}} \right)/{{\text{W}}_{{1}}}} \right]*100$$

The dinitrosalicylic acid (DNS) method was used to assess α-amylase activity as reported by Sadasivam and Manickam^32^. First, α-amylase was extracted from the seeds by graining them with 4 mL of 0.1 M CaCl_2_ in an ice bath, then centrifuging at 10,000 rpm for 20 min at 4 °C. The supernatant was collected and stored at 4 °C for α-amylase activity measurement. The amylase reaction was initiated by mixing 1 mL of supernatant and 1 mL of 1% soluble starch in acetate buffer solution (pH 5.5) and incubated at 37 °C for 15 min. After incubation, 1 mL of DNS reagent was added to stop the reaction. The mixture was then boiled at 100 °C for 5 min and cooled in an ice bath. Finally, the absorption at 560 nm was measured using a UV/Vis spectrophotometer (Thermo Fisher Scientific, USA) and the *α*-amylase activity calculated and reported in µmol maltose/min/g(FW).

#### Relative electrolyte leakage (REL)

Relative electrolyte leakage (REL) was evaluated in fresh seeds by the method of Singh et al.^33^ with modification. *A. tricolor* seeds (100 mg) were treated with emulsion and nanoemulsion at concentrations of 250 and 500 ppm, and Tween 80 solution at 500 ppm, by immersing them for a period of 6, 12, or 18 h. Deionized water served as a control. After the appropriate treatment, the seeds were dipped into 20 mL deionized water for another 24 h. Subsequently, the conductivity (EC_1_) of the samples was measured with a Consort C3010 multi-parameter analyzer (Consort, Belgium), then the dipped seeds were boiled for 20 min and the conductivity (EC_2_) measured again. REL was expressed as percentage and was calculated by the following formula:

$${\text{REL}}\% =\left( {{\text{E}}{{\text{C}}_{{1}}}/{\text{E}}{{\text{C}}_{{2}}}} \right)*100$$

### Statistics

A completely randomized design with four repetitions was used in all experiments. Data were expressed as mean value ± standard deviation (SD). Tukey’s multiple range test was used to compare the means (*p* < 0.05).

## Results and discussion

### Identification of *C. Odorata* EO constituents by gas chromatography/mass spectrometry (GC/MS)

**Fig. 2:**
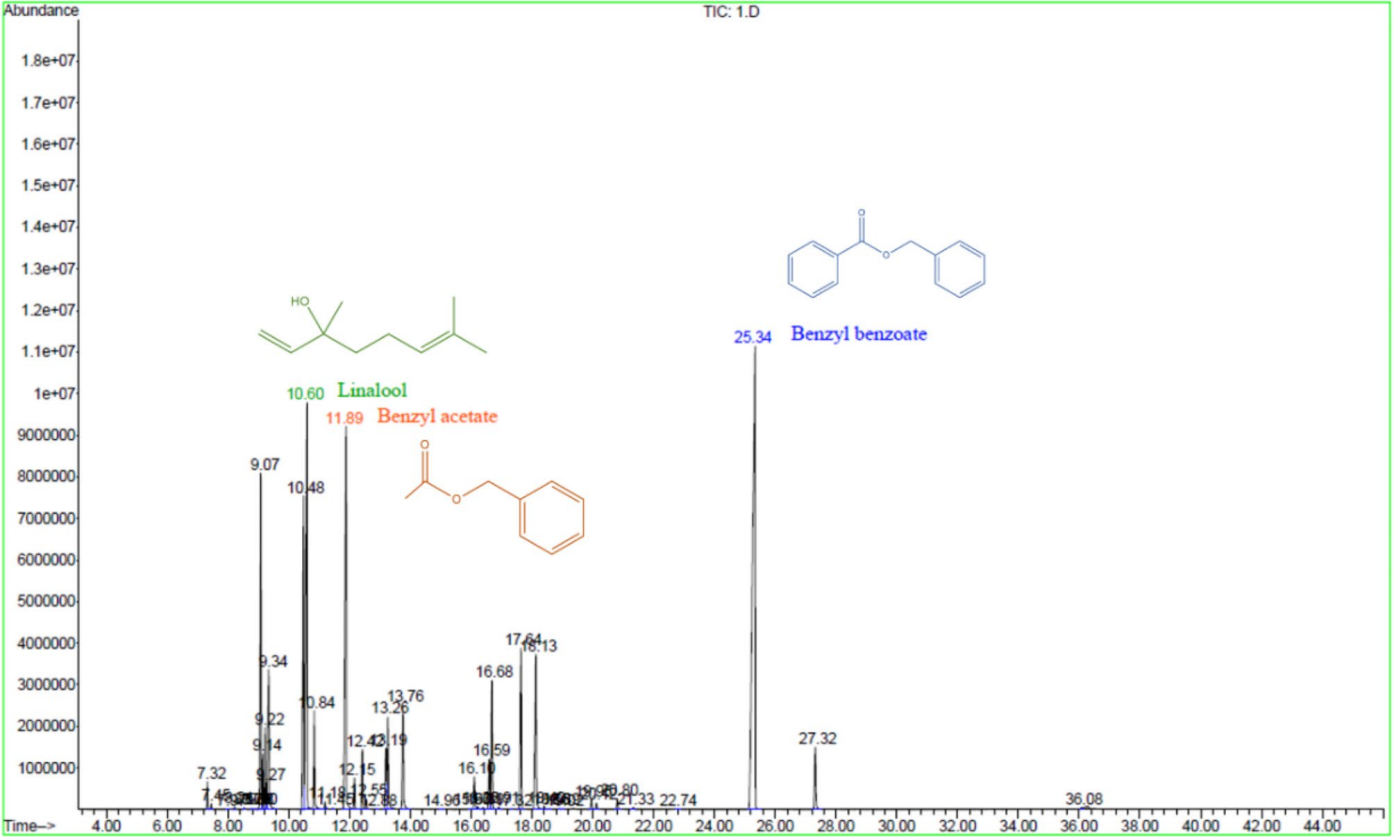
Gas chromatograph chromatogram of *C. odorata* EO.

*C. odorata* EO is a complex mixture of numerous chemical compounds. GC/MS analysis identified 25 different components constituting 98.224% of the total *C. odorata* EO. The major constituents were benzyl benzoate (27.117%), linalool (12.822%), and benzyl acetate (11.653%) (Table 1; Fig. 2). Previously, Acar et al.^34^ reported phytotoxic effects of benzyl benzoate on *Allium cepa* L, attributed to cell cycle effects and chromosomal damage. In terms of overall composition, the result obtained in this study is in good agreement with the report of Upadhyay et al.^8^, who determined that the main constituents of *C. odorata* EO were linalool (24.56%) and benzyl acetate (22.43%). Similary, Chakira et al.^35^ reported linalool and benzyl benzoate as the main constituents of *C. odorata* flower EO. The differences in these stated chemical compositions can be attributed to a variety of factors, including different harvest stages, genetic variants, climatic and environmental factors, and extraction procedures.

**Table 1:** Chemical constituents of essential oil from *C. Odorata*.

No.	Constituent	Formula	MW^a^	RT^b^	RI^c^	Peak area (%)
1	α-Thujene	C_10_H_16_	136.23	7.32	924	0.412
2	4-Methylanisole	C_8_H_10_O	122.16	9.074	1025	6.365
3	l-Limonene	C_10_H_16_	136.23	9.22	1030	1.246
4	1,8-Cineole	C_10_H_18_O	154.25	9.268	1031	0.358
5	Benzyl alcohol	C_7_H_8_O	108.14	9.338	1039	3.840
6	Methyl benzoate	C_8_H_8_O_2_	136.15	10.482	1076	7.346
7	Linalool	C_10_H_18_O	154.25	10.596	1100	12.822
8	Phenylethyl alcohol	C_8_H_10_O	122.16	10.838	1133	2.005
9	Benzyl acetate	C_9_H_10_O_2_	150.17	11.885	1166	11.653
10	4-Terpineol	C_10_H_18_O	154.25	12.155	1176	0.591
11	α-Terpineol	C_10_H_18_O	154.25	12.419	1189	1.250
12	γ-Terpineol	C_10_H_18_O	154.25	12.549	1193	0.216
13	Nerol	C_10_H_18_O	154.25	13.191	1228	1.800
14	Geraniol	C_10_H_18_O	154.25	13.757	1238	2.669
15	3-Phenyl-1-propanol	C_9_H_12_O	136.19	13.256	1242	2.021
16	Lavandulyl acetate	C_12_H_20_O_2_	196.29	16.682	1290	3.155
17	Eugenol	C_10_H_12_O_2_	164.20	16.104	1342	0.831
18	α-Copaene	C_15_H_24_	204.35	16.59	1380	1.157
19	Caryophyllene	C_15_H_24_	204.35	17.637	1404	4.196
20	*trans*-Caryophyllene	C_15_H_24_	204.35	20.119	1420	0.162
21	*trans*-Cinnamyl acetate	C_11_H_12_O_2_	176.21	18.133	1448	4.763
22	δ-Cadinene	C_15_H_24_	204.35	19.952	1523	0.249
23	*α*-Farnesene	C_15_H_24_	204.35	20.804	1739	0.256
24	Benzyl benzoate	C_14_H_12_O_2_	212.24	25.336	1762	27.117
25	Benzyl salicylate	C_14_H_12_O_3_	228.24	27.322	1876	1.744
					**Total**	**98.224**

^a^ = Molecular weight; ^b^ = Retention time; ^c^ = Retention index.

### Optimization of sonication amplitude and time

**Fig. 3:**
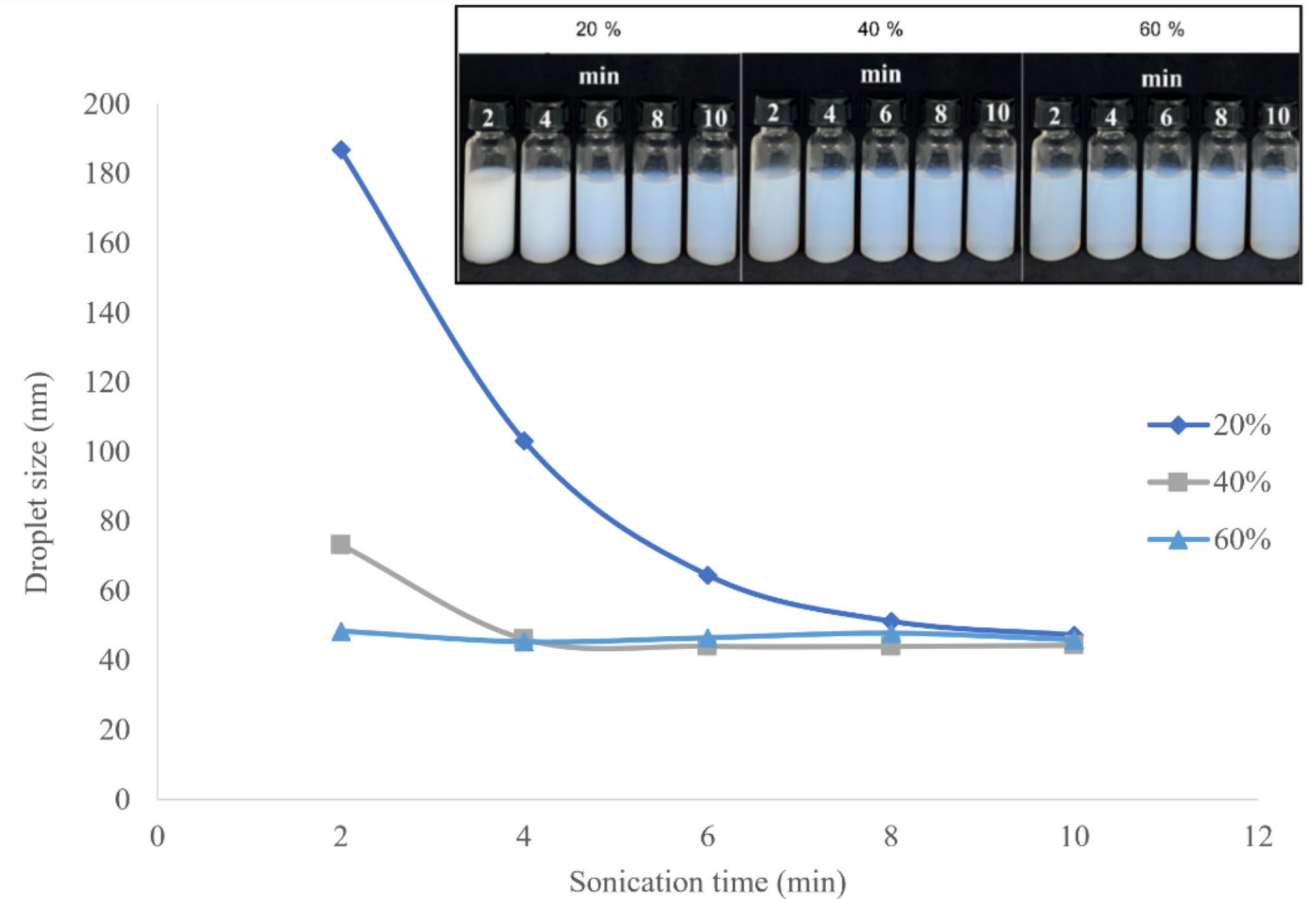
Effects of sonication amplitude (20–60%) and time (2–10 min) on nanoemulsion droplet size.

**Fig. 4:**
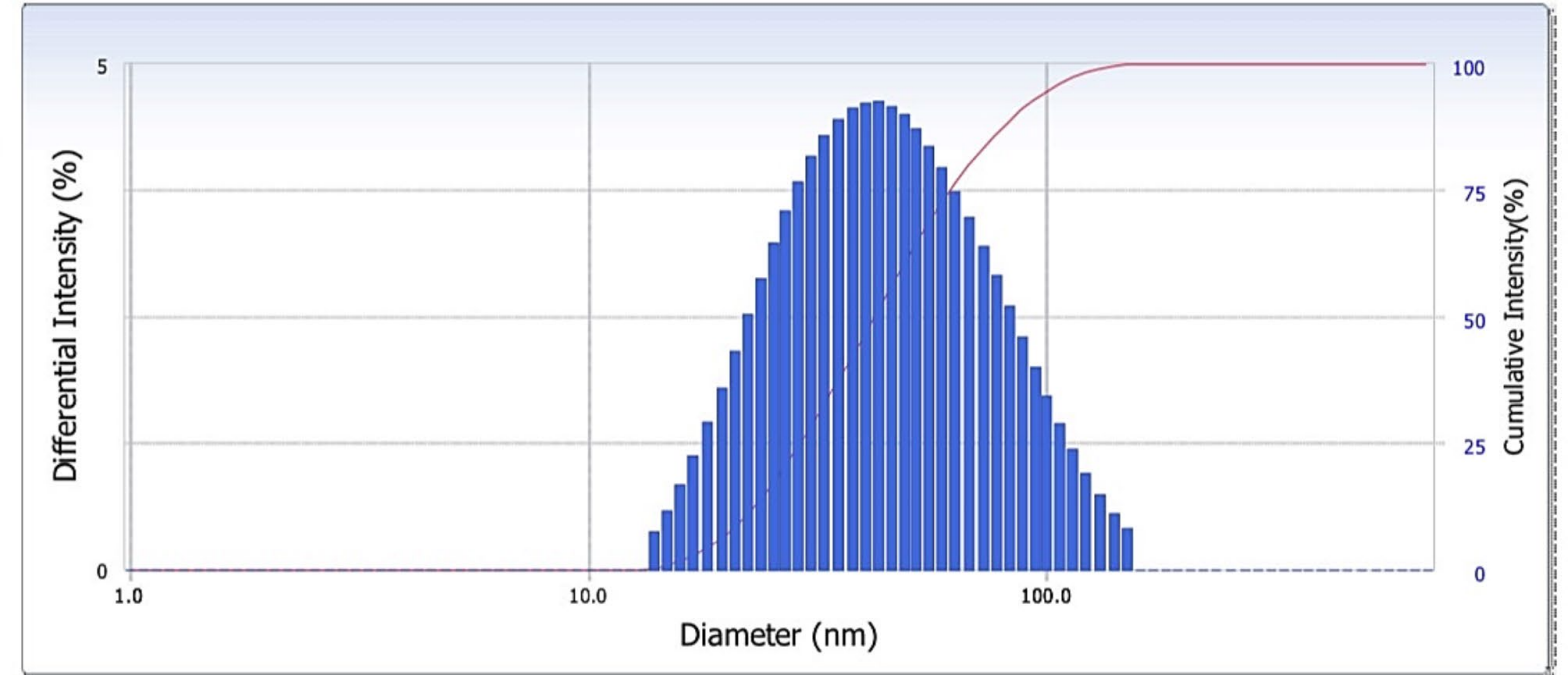
Intensity distribution of particle sizes for the optimized nanoemulsion (sonication amplitude 40% for 8 min), determined using dynamic light scattering.

The initial coarse emulsion had a milky-white visual appearance, with droplet size 855.4 ± 40.2 nm and PI value 0.478 ± 0.031 (data not shown). Large, aggregated emulsion droplets were present in the formulations generated without ultrasonication, which resulted in the largest droplet size (> 200 nm). Moreover, the high PI demonstrates the sample’s heterogeneity. In order to substantially disperse an oil phase in a continuous phase, significant energy input is needed to disrupt the oil-water interface^14^. Sonication is a convenient and well-researched method of providing the energy to convert particles to sub-micron size^36^. Here, an ultrasonic processor was used to develop the coarse emulsion of *C. odorata* EO into a nanoemulsion. In this method, shear forces created by ultrasonic cavitation cause large droplets to break up into smaller ones^14^. The line graph in Fig. 3 presents the droplet sizes of the nanoemulsions according to sonication amplitude (20–60%) and time (2–10 min). A longer sonication period is established to result in a higher net input energy, greater disruption of droplets, and greater reduction in droplet size^37^. The experimental results align with this trend, as nanoemulsion droplet size decreased with increasing sonication amplitude and time. Eight minutes of total sonication time with an amplitude of 40% was determined to provide the best size outcome of 43.98 ± 0.25 nm (Fig. 4); this formulation exhibited a blue tinge and no separated phase. Similarly, Carpenter and Saharan^14^ previously reported that shear forces produced by ultrasonic cavitation break apart bigger droplets into smaller ones. Further sonication did not result in substantially greater reduction of droplet size as determined by DLS. This can be explained by the fact that greater amplitude of energy release into the system significantly increases the cavitational collapse pressure at the end of asymmetric cavity collapse^14^. The longer sonication times result in increased interactions between the mechanical waves and the particles, yielding a higher rate of cavitation. At a certain point, droplet coalescence outperforms droplet breakup, which causes the particle size to increase^36^.

Table 2 shows the effects of sonication amplitude and sonication time on nanoemulsion PI value. The obtained PI values were less than 0.5, indicating a uniform droplet size distribution suitable for agricultural use^38^. The size distribution of a nanoemulsion system is considered narrow, and the formulation homogeneous, when the PI is less than 0.3^39^. Every formulation in this study had a PI value below 0.3 and therefore was homogeneous. This result supports that acoustic cavitation is effective in the production of nanoemulsions with small droplet size. Finding the optimal sonication time and amplitude is important because it affects the properties of the droplets. The optimal nanoemulsion was produced by amplitude of 40% for 8 min with smallest droplet size and selected for herbicidal activity experiments. This formulation was chosen to be investigated in further experiments.

**Table 2:** Effects of sonication amplitude and time on nanoemulsion PI value.

Sonication amplitude	Sonication time (min)				
	2	4	6	8	10
20	0.269 ± 0.006^ab^	0.280 ± 0.008^a^	0.275 ± 0.006^a^	0.253 ± 0.010^bc^	0.243 ± 0.006^cd^
40	0.276 ± 0.006^a^	0.224 ± 0.013^e^	0.219 ± 0.005^ef^	0.222 ± 0.006^ef^	0.225 ± 0.008^e^
60	0.228 ± 0.009^de^	0.205 ± 0.012^fg^	0.200 ± 0.007^g^	0.188 ± 0.007^g^	0.221 ± 0.007^ef^

Data presented as mean ± SD. Means with different letters are significantly different as indicated by Tukey’s test (*p* ≤ 0.05).

### Effect of surfactant fraction on droplet size and PI value of *C. Odorata* nanoemulsion

Nanoemulsion droplet size is significantly impacted by emulsifier concentration, here the surfactant Tween 80. In this study, Tween 80 fraction was varied from 0.5 to 3% w/v in formulations containing 1% w/v EO prepared at a sonication amplitude of 40% for 8 min. As the Tween 80 concentration increased from 0.5 to 1% w/v, the droplet size decreased gradually (Table 3). This decrease with increasing surfactant concentration occurs because the interfacial area is increased and interfacial tension decreased^36^. In addition to improving steric and electrostatic stabilization, the surfactant coating on the oil droplets produced Brownian motion, which stopped the molecules from interacting, thereby preventing the particles from coalescing and growing larger^36,40^. On the other hand, increasing Tween 80 beyond 1% w/v induced larger droplets (43.98 to 113.5 nm) because excess surfactant may lead to Ostwald ripening^41^.

**Table 3:** Droplet size and PI value of *C. Odorata* nanoemulsions incorporating different proportions of tween 80.

Parameter	C. odorata EO (%) : Tween 80 (%)			
	1:0.5	1:1	1:2	1:3
Size (nm)	97.0 ± 2.2^b^	43.98 ± 0.2^d^	78.6 ± 1.3^c^	113.5 ± 0.8^a^
PI	0.209 ± 0.010^c^	0.222 ± 0.006^c^	0.321 ± 0.007^b^	0.388 ± 0.009^a^

Data presented as mean ± SD. Means with different letters within a row are significantly different by Tukey’s test (*p* ≤ 0.05).

### Characterization of the optimized nanoemulsion

#### Fourier-transform infrared (FTIR) spectroscopic analysis

**Fig. 5:**
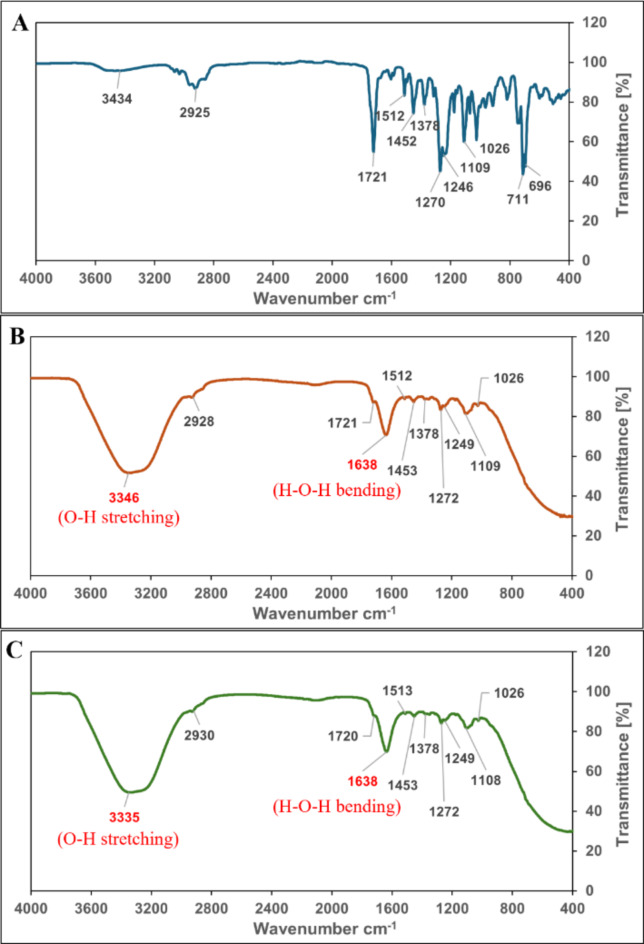
Fourier-transform infrared spectra of the 4000 –400 cm^-1^ region for *C. odorata* pure EO (**A**), coarse emulsion (**B**), and optimized nanoemulsion (**C**).

To confirm the non-destructive nature of the ultrasonic procedure, *C. odorata* pure EO (A), coarse emulsion (B), and the optimized nanoemulsion (C) were analyzed using an FT-IR spectrometer (Fig. 5). The pure EO exhibited typical bands at 3434 cm^− 1^ (O-H stretching), 2925 cm^− 1^ (aliphatic C-H stretching), 1721 cm^− 1^ (C = O stretching), and 1270 cm^− 1^ (C-O stretching), along with additional peaks at 1512, 1452, 1378, 1246, 1109, 1026, 711, and 696 cm^− 1^. Similar patterns were evident in the FT-IR spectra of the emulsion and nanoemulsion, which included most of the peaks observed for the pure EO. However, there were a few additional bands found, namely a very broad peak at 3346 cm^− 1^ (O-H stretching) and a peak at 1638 cm^− 1^ (H-O-H bending), both of which could be attributed to water. Thus, the FT-IR analysis proved that ultrasonic emulsification does not affect the structure of EO constituents.

#### Morphology

**Fig. 6:**
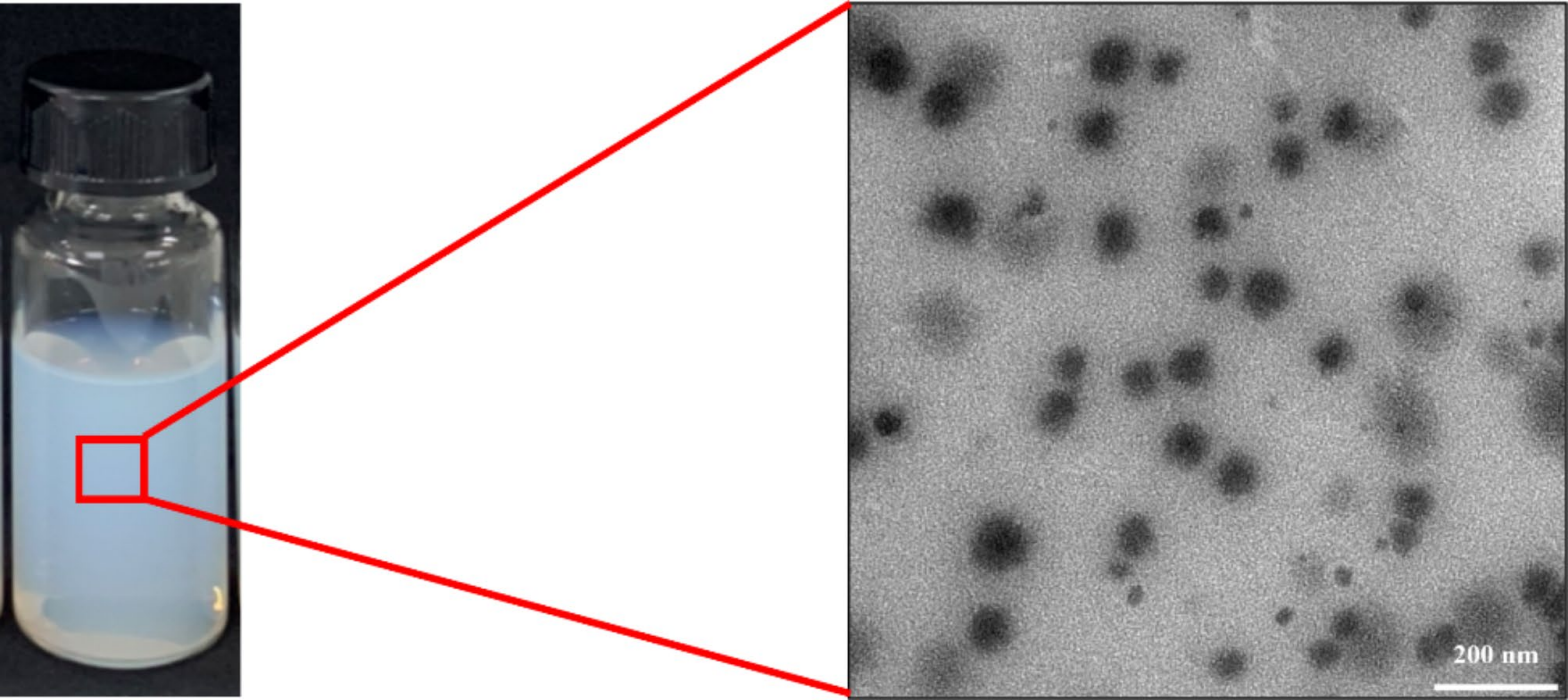
Representative TEM picture of the optimized *C. odorata* EO-based nanoemulsion. Scale bar represents 200 nm.

As DLS is an indirect method of droplet size determination based on light scattering and the Brownian motion of lipid droplets, it is frequently supplemented by direct observation of the lipid particles and system morphology through microscopy techniques^42^. Here, transmission electron microscopy (TEM) was used to examine droplet morphology and confirm the size determination by DLS. Figure 6 shows a representative TEM picture of the optimized *C. odorata* EO nanoemulsion, in which spherically shaped droplets with size below 200 nm are evident. This result is in agreement with Alam et al.^43^, who likewise observed spherical droplets in TEM images of a *C. odorata* nanoemulsion.

#### Rheological properties

**Fig. 7:**
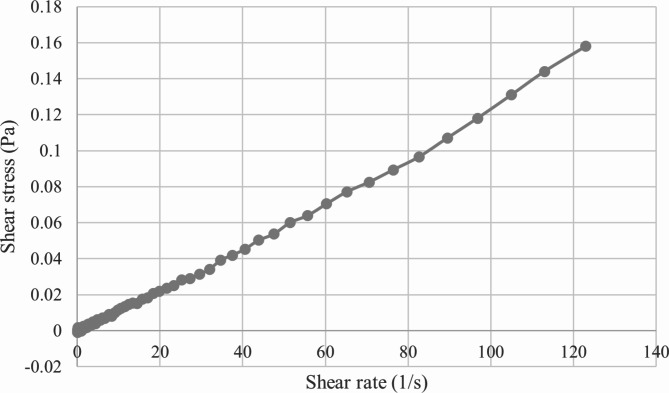
Flow curve of shear stress versus shear rate for the optimized *C. odorata* EO-based nanoemulsion.

Rheology is the scientific field that studies the flow and deformation of matter, and the rheological properties of nanoemulsions are important for many of their derivative products^44^. Figure 7 depicts the relationship between shear stress and shear rate in the optimized *C. odorata* EO-based nanoemulsion. The linear relationship of these properties indicated the most appropriate model for estimating the nanoemulsion’s flow behavior to be the Newtonian model^45^.

#### Surface tension

Surfactants are essential for maintaining the stability of a nanoemulsion system because they lower the interfacial tension and promote the production of small particles by absorbing at the oil-water interface. Tween 80’s high packing at the interface and its structure make it a potent solubilizer^46^. A low surface tension ensures optimal dispersion and blending of nanoemulsion constituents, potentially expanding the surface area over which bioactive ingredients and the recipient plant come into contact^22^. Moreover, low surface tension is crucial in pre-emergence herbicide formulations because it helps bioactive substances penetrate the hydrophobic seed surface. Here, the surface tension of the optimized *C. odorata* EO nanoemulsion was determined to be 33.53 mN/m. This low surface tension enables greater spreading, penetrating, and wetting capabilities, making it easier to apply the essential oil to plants as an agrochemical^36^.

#### Storage stability

**Fig. 8:**
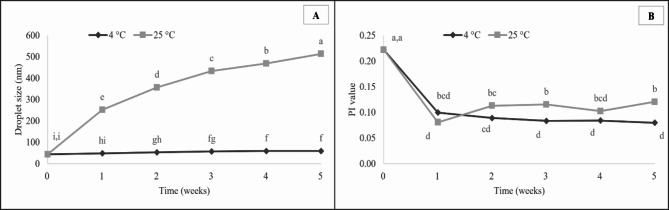
Droplet size (**A**) and PI value (**B**) of the optimized *C. odorata* EO-based nanoemulsion over five weeks of storage at 4 and 25 °C. Means with different letters are significantly different (*p* < 0.05).

We further investigated the relationship between nanoemulsion dispersion characteristics and storage stability. The line graph in Fig. 8 plots nanoemulsion droplet size and PI value over the course of 1–5 weeks of storage at 4 and 25 °C. After preparation and centrifugation, the optimal nanoemulsion showed no evidence of phase separation, indicating good stability against the force of gravitational separation. One of the key factors in determining herbicide efficacy, potency, and stability may be nanoemulsion preservation under ambient storage conditions^26^. Emulsion stability is influenced by temperature, which also affects the physical characteristics of its water, oil, and surfactant constituents^47^. Here, the droplet size of the optimized nanoemulsion changed only slightly when stored for five weeks at 4 °C. On the other hand, storage at 25 °C resulted in droplet size increasing over time (from 43.98 to 513.76 nm). The Ostwald ripening mechanism, which predominates in the destabilization of nanoemulsions, caused droplets to coalesce and form bigger droplets during storage^48–51^. Our result indicates that temperature significantly affects nanoemulsion droplet size over time, and also other associated characteristics, including PI value. Specifically, PI value significantly decreased in the first week, then changed slightly from weeks 1 to 5 for both storage temperatures of 4 and 25 °C. During storage, the nanoemulsion oxidizes gradually, and the degree of oxidation increases with temperature and storage time. Lipid oxidation may alter the nanoemulsion’s interfacial composition, which could lead to the emulsifier rearranging and desorbing at the interface and affecting the emulsion system’s stability^52,53^. The optimal clove EO-based nanoemulsion should store at 4 °C at least five weeks with nanodroplet size. All told, our findings suggest that temperature is a key determinant of the storage stability of a *C. odorata* EO-based nanoemulsion.

### Herbicidal activities

#### Seed germination and seedling growth

Table 4 compares the germination inhibition effects of the *C. odorata* EO-based coarse emulsion and optimized nanoemulsion. Overall, the inhibition effect increased with increasing concentration; however, the nanoemulsion had greater effect. When applied at a concentration of 250 ppm, the nanoemulsion completely inhibited seed germination while the coarse emulsion achieved only 63.75% inhibition over control. Likewise, after seven days of incubation, the nanoemulsion showed stronger inhibition of seed germination and seedling growth compared to the same concentration of coarse emulsion. This may be attributed to the tiny particle size facilitating easy penetration into the membranes of the seeds^36^. Previous research has demonstrated that EOs have good dose-dependent inhibitory potential against both radical elongation and germination^6^; however, ultrasonic formulations including an EO and emulsifier improve solubility and penetration into plant tissues^7^. Apart from particle size, two other properties that restrict the inhibitory potential of oil constituents are evaporation and oxidation^6,36,47^. In this respect, it is important to ensure efficient release and quick contact of active compounds with plant cells following application to weed seeds. Increased efficacy of a *Foeniculum vulgare* EO nanoemulsion over the base EO against *Triticum aestivum* has been reported by Kaur et al.^36^, attributed to improved essential active chemical and surface characteristics, efficient active ingredient release, and rapid plant cell interaction.

**Fig. 9:**
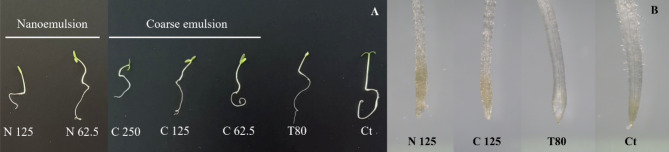
Morphology of *A. tricolor* seedling (**A**) and root (**B**) after treatment with *C. odorata* EO-based emulsion and optimized nanoemulsion. Ct = control (water); T80 = Tween 80 solution; C 62.5, C 125, C2 50 = coarse emulsion at 62.5, 125, and 250 ppm; N 62.5 and N 125 = nanoemulsion at 62.5 and 125 ppm.

A dose-dependent pattern of decreased shoot and root length was also observed for both the coarse emulsion and nanoemulsion, with effective inhibition of seedling growth at seven days after treatment with high doses (Table 4; Fig. 9A). Figure 9B illustrates the effects of 125 ppm emulsion and nanoemulsion on the morphology of *A. tricolor* root tips. Overall, both formulations might change the root tip structure, resulting in root tip damage, root elongation, or inhibition of water absorption. Root hairs were observed only in the control treatment (water). In previous reports, changes in root shape have been noted in response to a number of phytochemicals, including the polyphenol rosmarinic acid and the sesquiterpene hydrocarbon farnesene^54^.

The optimal nanoemulsion completely inhibited the seed germination and seedling growth of *A. tricolor* at concentration of 250 ppm. All told, comparison of the coarse emulsion and nanoemulsion revealed that plants treated with the nanoemulsion showed more injury, structure alteration, and root tip damage than those treated with the coarse emulsion. Our results indicated that *C. odorata* EO-based nanoemulsion with ultrasonication exhibited superior weed control efficacy at lower concentrations compared to conventional coarse emulsion. However, our study focused primarily on laboratory evaluations of herbicidal efficacy. Therefore, the practical effectiveness of the nanoemulsion in many agricultural applications, including variability in weed species, soil types, and climate, still need further investigation.

**Table 4:** Inhibitory effect of *C. odorata* EO-based coarse emulsion and optimized nanoemulsion on seed germination and seedling growth of *A. Tricolor*.

Treatment	Concentration	Inhibition (% over control)		
	(ppm)	Germination	Shoot length	Root length
Tween 80 solution	500	3.75 ± 0.50^f^	5.14 ± 1.82^e^	4.46 ± 1.24^d^
Coarse emulsion	62.5	13.75 ± 4.79^e^	25.51 ± 3.83^d^	4.75 ± 0.58^d^
	125	28.75 ± 6.29^d^	32.30 ± 2.27^cd^	12.37 ± 1.72^d^
	250	63.75 ± 2.50^b^	40.12 ± 2.46^bc^	40.00 ± 2.32^b^
	500	96.25 ± 4.79^a^	91.26 ± 10.13^a^	90.65 ± 10.86^a^
Nanoemulsion	62.5	16.25 ± 2.50^e^	29.84 ± 1.82^d^	5.90 ± 0.74^d^
	125	47.50 ± 2.89^c^	47.94 ± 3.11^b^	25.32 ± 3.53^c^
	250	100 ± 0^a^	100 ± 0^a^	100 ± 0^a^
	500	100 ± 0^a^	100 ± 0^a^	100 ± 0^a^

Data presented as mean ± SD. Means with different letters within a column are significantly different by Tukey’s test (*p* ≤ 0.05).

#### Seed imbibition

**Fig. 10:**
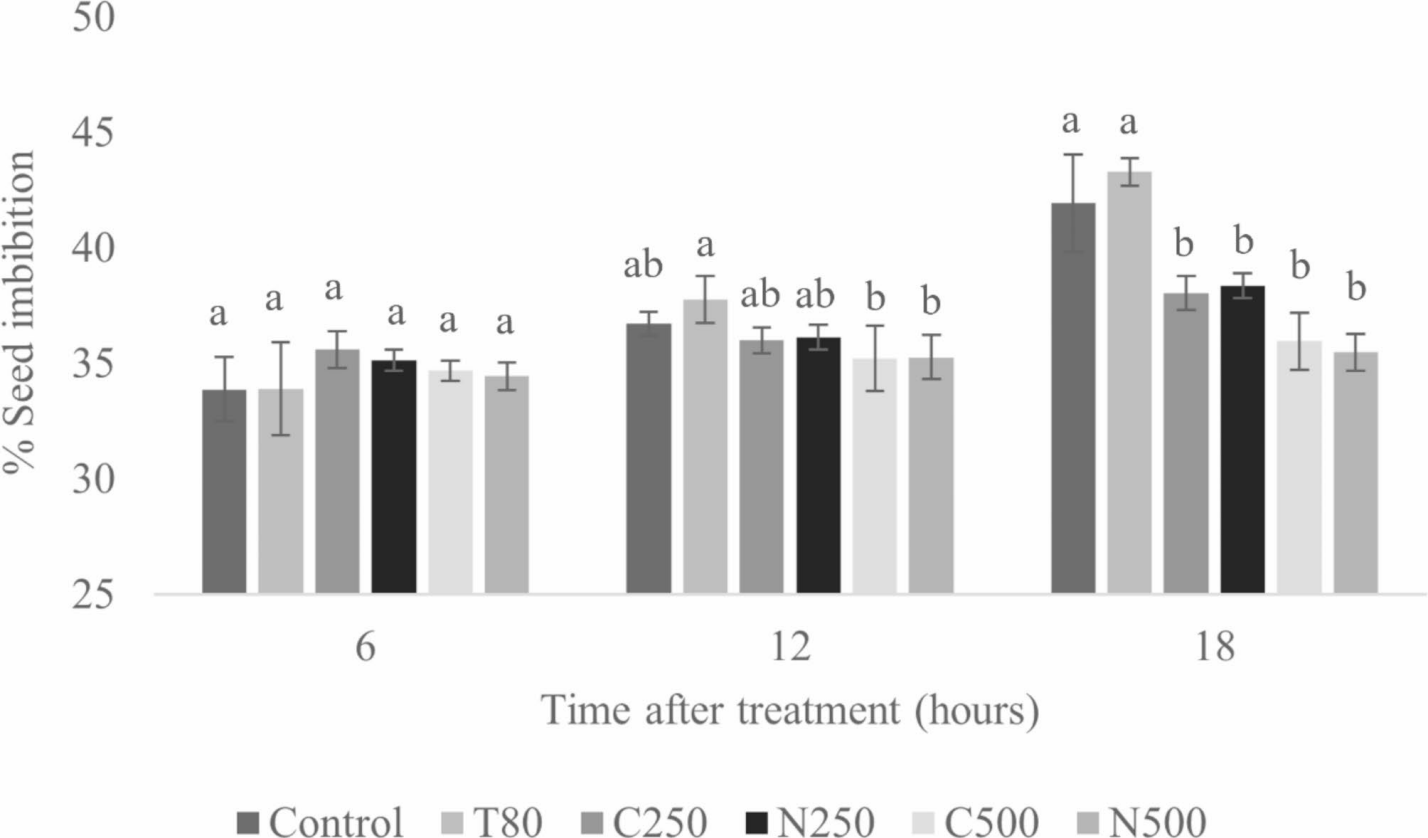
Effect of the *C. odorata* EO-based coarse emulsion and optimized nanoemulsion on seed imbibition of *A. tricolor* seeds at 6, 12, and 18 h. T80 = Tween 80 solution; C250 and C500 = coarse emulsion at 250 and 500 ppm; N250 and N500 = nanoemulsion at 250 and 500 ppm. Error bars represent standard deviation.

The process of seed germination begins with seed imbibition. Figure 10 shows the effect of the coarse emulsion and nanoemulsion on seed imbibition of *A. tricolor*. With increasing imbibition period (from 6 to 18 h), imbibition in the control samples (water and Tween 80) increased progressively, while seeds in coarse emulsion or nanoemulsion showed much less imbibition. The nanoemulsion treatment resulted in the lowest percentage of seed imbibition due to seed membrane damage causing membrane disruption and permeability. The seed coat normally has the potential to have selective permeability and thereby protects the seed from dangerous outside factors. Penetrating the seed coat is therefore necessary to interfere with the germination process. Lower surface tension increases the spreading and wetting characteristics of a formulation, resulting in a decreased contact angle^46^. For this reason, a surfactant is included in herbicide formulations not only to help stabilize the system but also to boost its ability to absorb into the seed coat. Similarly, these outcomes are consistent with findings from the application of citronella EO-based nanoemulsion to *E. crus-galli* seeds, which showed that the inhibitory effect on imbibition increased with increasing EO concentrations.

#### α-Amylase activity

**Fig. 11:**
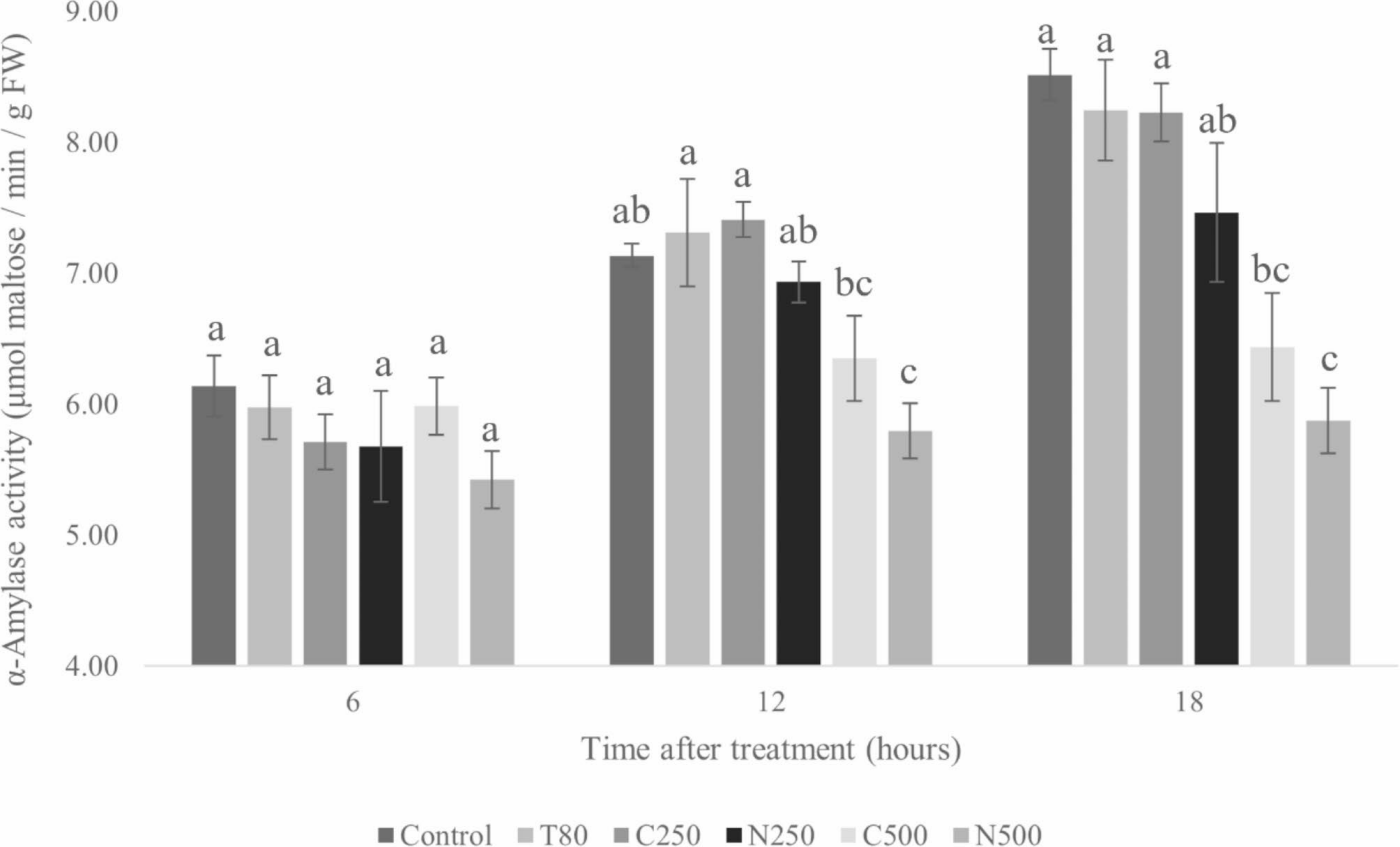
Effect of the *C. odorata* EO-based coarse emulsion and optimized nanoemulsion on α-amylase activity of *A. tricolor* seeds at 6, 12, and 18 h after treatment. T80 = Tween 80 solution; C250 and C500 = coarse emulsion at 250 and 500 ppm; N250 and N500 = nanoemulsion at 250 and 500 ppm. Error bars represent standard deviation.

The bar graph in Fig. 11 shows the effect of the *C. odorata* EO-based coarse emulsion and nanoemulsion on α-amylase activity and carbohydrate degradation in seeds. Following imbibition, catabolic mechanisms initiate seed germination^4^. α-Amylase breaks down starch into smaller organic molecules during the germination process, which provides the energy and nutrients required for germination^47^. Here, activity of α-amylase was measured at 6, 12, and 18 h after treatment. Overall, as time increased, α-amylase activity rose in all samples. In the first six hours, seeds treated with either emulsion did not exhibit a significant difference from the control. After 12 and 18 h, however, there was a notable reduction in α-amylase activity, dependent on the emulsion dose. This effect could be the cause of the observed inhibition in seed germination. Across all concentrations and times, the nanoemulsion performed better than the same concentration of coarse emulsion, although the difference did not achieve significance. Additionally, seeds treated with nanoemulsion at the concentration of 500 ppm exhibited the lowest α-amylase activity, which is in line with the imbibition assay findings. All told, these results indicate the nanoemulsion to potentially effect more potent inhibition of α-amylase activity than the coarse emulsion. As such inhibition prevents starch from being broken down into small molecules to support growth and development, this effect may ultimately result in suppression of seed germination and seedling growth.

#### Membrane integrity

**Fig. 12:**
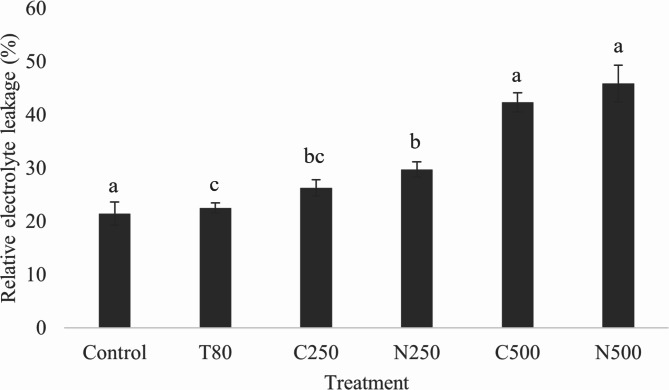
Effect of the *C. odorata* EO-based coarse emulsion and optimized nanoemulsion on membrane electrolyte leakage of *A. tricolor* seeds at 18 h after treatment. T80 = Tween 80 solution; C250 and C500 = coarse emulsion at 250 and 500 ppm; N250 and N500 = nanoemulsion at 250 and 500 ppm. Error bars represent standard deviation.

**Fig. 13:**
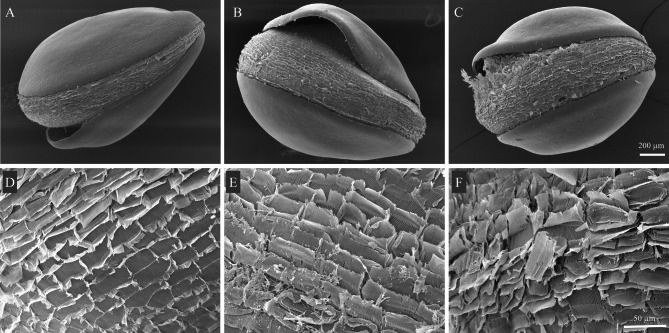
Scanning electron microscope images showing the physical effect of *C. odorata* EO-based emulsion and optimized nanoemulsion treatments (250 ppm) on *A. tricolor* seeds. Scale bar represents 250 μm (**A**-**C**) and 50 μm (**D**-**F**).

The effect of the coarse emulsion and nanoemulsion on seed membrane integrity were evaluated in terms of the relative electrolyte leakage (REL). % REL is an indicator of membrane leakiness in that increased electrical conductivity occurs as a result of damaged membrane integrity^36^. Destabilization of the plant plasma membrane can result in cell death because the membrane is essential for preserving cellular integrity^55^. Figure 12 shows the effect of the coarse emulsion and nanoemulsion on membrane electrolyte leakage of *A. tricolor* seeds at 18 h after treatment. Overall, % REL increased with increasing concentration of either emulsion, with the highest % REL (45.85%) achieved by the nanoemulsion at 500 ppm. Both emulsions cause greater permeability and electrolyte leakage when compared to the control, as they compromise cell membrane integrity. These findings agree with previous observations that monoterpenes found in essential oils can cause ion leakage from plant tissues^6,12,36,56^. Compared to the coarse emulsion, the nanoemulsion resulted in higher REL values, although the difference did not achieve significance. Previous reports support nanoemulsion treatments producing higher REL values than coarse emulsions on account of their minuscule droplet size and easy penetration into the seed membrane^36^. The observed trend in REL may correlate with the scanning electron microscopy imaging (Fig. 13), which revealed the greatest tissue damage to occur in nanoemulsion-treated seeds.

## Conclusion

In this study, GC/MS analysis indicated *C. odorata* EO to be mainly composed of benzyl benzoate (27.117%), linalool (12.822%), and phenylmethyl ester (11.653%). Subsequently, EO-based nanoemulsions were formulated using the ultrasonic emulsification method with Tween 80. It was observed that the smallest droplet size (43.98 nm, PI 0.222) was produced at a sonication amplitude of 40% for 8 min. FT-IR confirmed that the ultrasonication method did not degrade the components of the EO. Assessment of the herbicidal efficacy of the optimized nanoemulsion against *A. tricolor* revealed it to exert strong phytotoxic effects on seed germination, seed membrane integrity, and plant development, more so than the coarse emulsion. These findings support the use of *C. odorata* EO-based nanoemulsions as a natural herbicidal product for sustainable weed management. Furthermore, in order to fully utilize the benefits of the *C. odorata* EO-based nanoherbicide, this research is necessary to evaluate how the nanoemulsion may be used to control weeds in various types of field conditions, including the scalability of production techniques to ensure cost-effectiveness for broad agricultural application.

## Data Availability

All data generated or analyzed during this study are included in this published article.
